# Closed-loop management of inpatient hyperglycemia

**DOI:** 10.18632/aging.102144

**Published:** 2019-08-08

**Authors:** Charlotte K. Boughton, Lia Bally, Roman Hovorka

**Affiliations:** 1 Wellcome Trust-MRC Institute of Metabolic Science, University of Cambridge, Cambridge, UK; 2 Wolfson Diabetes and Endocrine Clinic, Cambridge University Hospitals NHS Foundation Trust, Cambridge, UK; 3 Department of Diabetes, Endocrinology, Clinical Nutrition & Metabolism, Bern University Hospital, Bern, Switzerland

**Keywords:** hyperglycaemia, inpatient management, frailty, diabetes technology, closed-loop glucose control

The prevalence of diabetes in the hospital is increasing and approximately 18-20% of hospital beds are occupied by someone with diabetes [1]. Diabetes disproportionally affects the elderly, with three times greater prevalence in hospitalised people aged over 65 years than in those aged under 45 years [2]. Maintaining near normoglycaemia during hospital admissions can be very challenging. The impact of the current illness, medication changes, alterations to meal timings and intake, and requirement for nutrition support in hospital can all contribute to sub-optimal glucose control. Both hyper- and hypoglycemia in hospital are associated with increased risk of complications, length of stay, admission to the intensive care unit and mortality [3].

Attempts to attain target glucose levels with current insulin therapy (multiple daily subcutaneous insulin injections) titrated according to capillary blood glucose measurements can increase the risk of hypoglycemia and increase workload for healthcare professionals [4]. Older adults are more prone to hypoglycaemia due to higher prevalence of comorbidities including polypharmacy, renal and hepatic failure, malnutrition, cognitive impairment and frailty. Additionally, counter-regulatory mechanisms to hypoglycaemia may be attenuated in older adults and symptoms less marked.

**Figure 1:**
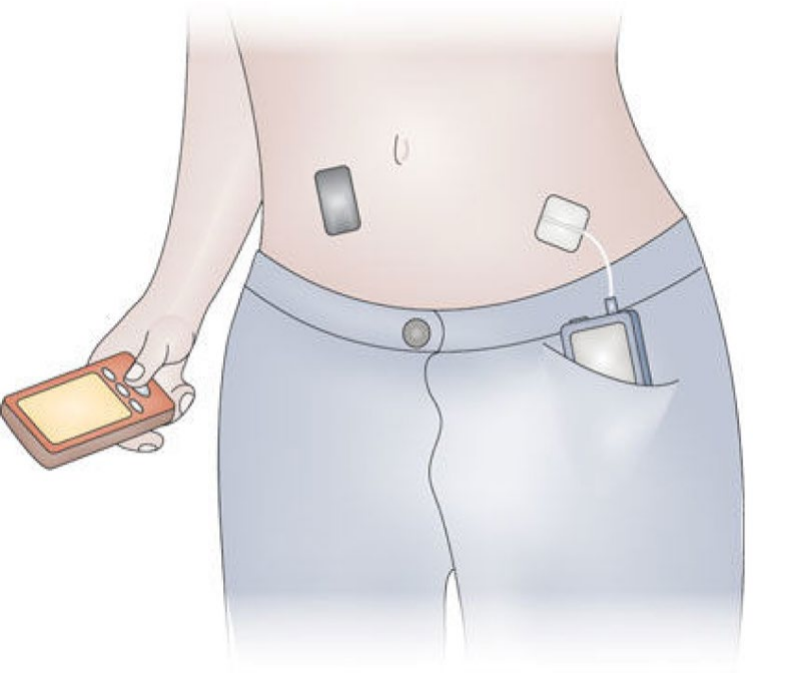
**Closed-loop system.** A continuous glucose monitor (black device on abdomen) transmits information about interstitial glucose levels to a smartphone (in the hand), hosting a control algorithm. An insulin pump (in the pocket) delivers a rapid-acting insulin analogue subcutaneously. Insulin delivery is modulated in real-time by the control algorithm. Communication between system components is wireless.

The significant advances in diabetes technologies, including subcutaneous insulin pumps and continuous glucose monitoring (CGM) systems, over the past 10 years have led to the development of automated insulin delivery (closed-loop) systems. The closed-loop approach involves communication of real-time glucose data provided by a CGM device, to a control algorithm (hosted on a smartphone or the insulin pump), which then instructs subcutaneous insulin delivery via the insulin pump, automatically modifying the insulin infusion rate every 5-10 minutes based on the sensor glucose levels (Figure 1). One of the key benefits of closed-loop glucose control is the automatic and continuous modulation of insulin delivery rates to adapt to within day and between day variability of exogenous insulin requirements.

Hybrid closed-loop systems require the user to manually initiate mealtime insulin boluses, while fully closed-loop systems automatically dose insulin without information about meals with the advantage of reduced user interaction.

Hybrid closed-loop systems have been approved by regulatory authorities for people with type 1 diabetes and are increasingly being used. So far, no closed-loop system has been approved for the management of inpatient hyperglycaemia but we believe that such systems are feasible and may provide substantial benefits as described in this editorial.

The feasibility of fully automated closed-loop insulin delivery without meal announcement in the inpatient setting has been demonstrated to be safe and effective in achieving near-normal glucose control [5].

A randomised controlled trial involving 136 adults on the general wards with hyperglycaemia compared fully closed-loop insulin delivery with standard insulin therapy for a period of up to 15 days or until discharge [6]. The time spent with glucose in the target range (100-180 mg/dL) was 66% with closed-loop compared with 42% with standard insulin therapy. This 24 percentage point increase with closed-loop equates to almost six additional hours each day with glucose levels in target range. This was achieved without any increase in hypoglycemia (<54 mg/dL) or in the amount of insulin delivered compared to conventional therapy. Closed-loop insulin delivery was associated with superior glycaemic control without a higher risk of hypoglycemia.

Determining those most likely to benefit from closed-loop insulin delivery is critical to support widespread adoption of this technology, and demonstrating efficacy and safety of closed-loop in the more challenging inpatient sub-populations is important for generalisability.

Glucose management in people with diabetes receiving hemodialysis can be demanding due to high day to day variability in glucose levels and insulin requirements. A subgroup analysis of the study by Bally et al [6] examined the impact of fully closed-loop insulin delivery in patients undergoing hemodialysis while in hospital, compared with standard insulin therapy [7]. Those using closed-loop spent significantly more time with glucose levels in target range than the control group (69% vs 32% respectively), without increasing the risk of hypoglycemia. The closed-loop approach may offer a novel approach to manage diabetes in this vulnerable population and further outpatient studies are warranted.

Administration of nutrition support (enteral/parenteral nutrition) in the inpatient setting is associated with hyperglycaemia in up to 50% of patients. Management of feed-induced hyperglycemia with standard insulin therapy can be complex due to unanticipated interruptions to nutrition support and frequent changes to feeding regimens demanding considerable input by healthcare professionals. In a randomised controlled study involving 43 inpatients receiving nutritional support, fully automated closed-loop insulin delivery using faster-acting insulin aspart was associated with superior glucose control compared to standard insulin therapy [8]. The closed-loop group spent approximately eight additional hours each day with glucose levels in target range (100-180 mg/dL) compared with those receiving standard insulin therapy (68% v 36%) without an increase in hypoglycemia. Fully automated insulin delivery is a safe and effective tool to improve glycaemic control in hospitalised patients receiving nutritional support, where glucose management can be particularly challenging.

The closed-loop approach is an attractive option to positively change the management of inpatient diabetes particularly in the most challenging inpatient populations; larger studies are required to determine if the observed improved glucose control with closed-loop insulin delivery can translate into improved clinical outcomes for patients, and reduce staff work burden and the healthcare costs associated with inpatient diabetes.
